# Another Look Ahead

**Published:** 1883-06

**Authors:** 


### ANOTHER LOOK AHEAD.

The May number of the INDEPENDENT PRACTITIONER was issued from Buffalo, and we then intimated a change of ownership, but were unable to give the results of negotiations then in progress. Nor are we yet prepared to announce all the good things which are in store for our dental readers.

Experience has taught that a journal like this must have a definite aim, and can satisfactorily serve but one class of readers. The general practitioner demands reading matter especially intended for and adopted to his wants, and the specialist desires the same. We have the very highest authority for the declaration that one cannot serve two masters: "Either he will hate the one and love the other, or else he will hold to the one and despise the other." Dentistry has become too great to be sufficiently served by a mere journalistic department. There is a large class of diseases which have too often been placed under the care of the general practitioner, but which the intelligent and educated dentist is most competent to treat. A general view of these conditions should be of interest to every medical man, but their particular consideration ought to be from the standpoint of the dentist. Therefore, it has been deemed advisable, at the close of this volume, to devote the INDEPENDENT PRACTITIONER to the specialty of Dentistry.

A dental journal, to meet all the wants of the profession, ought, of right, to be conducted by dentists; but the successful practitioner has usually little time for matters outside his office, and were one man to edit and publish a creditable journal it would demand his retirement from active practice, and thus defeat one of the objects in view.

Again, it is a notorious fact that the profession is slow to support any enterprise which has for its aim only the general good, and hence a paying journal, it has been supposed, must have the financial support of some mercantile interest. Yet there are a few dentists who are willing to spend and be spent for the love of the profession to which they have dedicated their lives. They believe that dentistry should have some journals which are not hampered by any necessity for mere money getting; that there are some important interests which may be best served by a professional magazine that is disconnected from any commercial enterprise. A burthen which would be too grievous for any one man may be easily borne by the co-operation of a number. Therefore, when it was found that the INDEPENDENT PRACTITIONER must change hands, a meeting of dentists was held, and an agreement to undertake the work of its publication was made. The first plan suggested was to engage in the enterprise representative men from every section of the United States, but further consideration revealed the impracticability of this scheme, because meetings for necessary consultation and counsel would be impossible. It was, therefore, determined to confine the active management and risk of the undertaking to a comparatively small number of men, who could more easily be got together for any necessary conference, and to ask the co-operation of the others without imposing upon them unnecessary labor.

That association has been formed, and this journal is now under their control. It will be published by them without any expectation of pecuniary return. If it pays its way they will be abundantly satisfied, and should it do more they will devote any surplus, after paying necessary expenses, to internal improvements; adding desirable illustrations, offering payment for original articles, and increasing its size. It hopes for the active support of every dentist who loves his profession, and it asks for sympathy in its disinterested work. It especially appeals to the writers in the profession for assistance, and believes that it will be enabled to lay their communications before the thinking men in dentistry, and thus secure a full consideration of anything which they may desire to present. It invokes the assistance of every dentist in the removal of the reproach of the profession, that no sympathy or support will be given to any one who labors from disinterested motives.

The following dentists have embarked in this undertaking, and each one pledges himself that his very best efforts shall be exerted to make the INDEPENDENT PRACTITIONER a credit to dentistry, and worthy the support of the profession:

Frank Abbott, New York.
W. C. Barrett, Buffalo.
C. F. W. Bodecker, New York.
William Carr, New York.
C. E. Francis, New York.
O. E. Hill, Brooklyn.

Wm. Jarvie, Jr., Brooklyn.
A. L. Northrop, New York.
S. B. Palmer, Syracuse.

The executive committee, who will have charge of all the business affairs of the Journal, is composed of Drs. Carr, Abbott and Hill. The Treasurer is Dr. C. E. Francis. The Editor will be Dr. W. C. Barrett.

All communications, therefore, which relate to business, such as subscriptions, advertisements, and matters of like nature, should be directed to Dr. William Carr, 35 West 46th Street, New York.

Editorial communications, exchanges, books for review, and articles for editorial notice, must be sent to Dr. W. C. Barrett, No. 11 West Chippewa Street, Buffalo, New York.

